# Impact of malaria on glutathione peroxidase levels: a systematic review and meta-analysis

**DOI:** 10.1038/s41598-023-41056-x

**Published:** 2023-08-25

**Authors:** Manas Kotepui, Aongart Mahittikorn, Nsoh Godwin Anabire, Kwuntida Uthaisar Kotepui

**Affiliations:** 1 Medical Technology, School of Allied Health Sciences, Walailak University, Tha Sala, Nakhon Si Thammarat, Thailand; 2 Department of Protozoology, Faculty of Tropical Medicine, Mahidol University, Bangkok, Thailand; 3 Department of Biochemistry and Molecular Medicine, School of Medicine, University for Development Studies, Tamale, Ghana; 4 West African Centre for Cell Biology of Infectious Pathogens (WACCBIP), Department of Biochemistry, Cell and Molecular Biology, University of Ghana, Accra, Ghana

**Keywords:** Biomarkers, Malaria

## Abstract

The primary antioxidant, glutathione peroxidase (GPx), is hypothesized to contribute to the pathophysiology of malaria. This current study conducted a meta-analysis to examine variations in GPx blood levels in malaria patients. Seven electronic databases—ProQuest, Scopus, Embase, MEDLINE, PubMed, Ovid, and Google Scholar—were searched for relevant studies with no limitations to publication language or publication date. The Joanna Briggs Institute critical appraisal tools were used to appraise the risk of bias among the included studies critically. The meta-analysis was conducted by pooling the effect estimates and Hedges’s g using a random-effects model. Search results returned 1253 articles, of which 16 studies were used for syntheses. Results of the meta-analysis indicated that malaria patients had decreased blood levels of GPx compared to uninfected individuals (*P* < 0.01, Hedges’ g: − 4.06, 95% CI − 5.49–(− 2.63), *I*^2^: 99.07%, 1278 malaria patients/627 uninfected individuals, 15 studies). Subgroup analyses indicated that peripheral levels of GPx were significantly diminished in patients with *P. falciparum* malaria compared to uninfected controls (*P* < 0.01, Hedges’ g: − 3.06, 95% CI − 4.46–(− 1.65), *I*^2^: 98.39%, 9 studies) but not in patients with *P. vivax* malaria (*P* = 0.15, Hedges’ g: − 2.05, 95% CI − 4.83–0.74), *I*^2^: 98.64%, 2 studies) Overall, malaria is associated with declined levels of GPx, particularly in patients with *P. falciparum* malaria. The finding provides valuable insights that prompt the need to investigate the role of GPx depletion in malaria pathogenesis.

## Introduction

Five different *Plasmodium* parasite species cause human malaria, namely *Plasmodium falciparum*, *Plasmodium vivax*, *Plasmodium ovale*, *Plasmodium malariae*, and *Plasmodium knowlesi*, with *P. falciparum* being the most dangerous and prevalent in Africa^1^. *P. vivax*, a different species of *Plasmodium*, has been implicated in a growing number of severe illnesses, especially in vulnerable populations such as young children and pregnant women^2^. In southeast Asia*, P. knowlesi* infection significantly contributes to zoonotic malaria^3^. Malaria continues to spread despite tremendous efforts to control and eradicate it, with 247 million cases and 625,000 deaths reported in 84 countries in 2021^1^.

Host-related factors such as genetic polymorphisms, immune responses, and antioxidants play key roles in malaria pathogenesis and related clinical signs and symptoms^4–6^. During *Plasmodium* infections, both the host and parasite encounter oxidative stress due to increased production of reactive oxygen species (ROS). ROS, such as the superoxide anion and hydroxyl radical, can be produced by activated neutrophils and macrophages in the host and by degrading parasitic hemoglobin^6,7^. *Plasmodium* infections in erythrocytes can lead to the degradation of host hemoglobin, which plays a pivotal role in generating oxidative stress^8^. In addition to the oxidative stress resulting from metabolic processes, the host immune system’s production of ROS further contributes to the overall oxidative burden experienced by the parasitized cell^8^. The large amount of ROS generated by hemoglobin degradation and the host immune system has been associated with severe malaria and a higher mortality rate^9^.

The relationship between oxidative stress and antioxidant levels is an essential host-related factor as depletion of antioxidants, such as reduced glutathione (GSH), antioxidant vitamins (A, C, and E), and superoxide dismutase (SOD), leads to increased severity of malaria^10–14^. Glutathione peroxidase (GPx) is a member of the oxidoreductase family that catalyzes the conversion of hydrogen peroxide and organic hydroperoxides into water or related alcohols^15^. GPx works with catalase (CAT) and SOD to form an enzymatic antioxidant system which is the first-line antioxidant defense system^16^. The role of GPx in malaria is controversial because GPx is absent from *P. falciparum*^17^. Previous studies have reported decreased levels of GPx in malaria^18,19^. In contrast, other studies have shown contrasting results; levels of GPx were either elevated or comparable in malaria cases relative to uninfected individuals^20,21^. As the levels of GPx in malaria are inconsistent, and the role of GPx in malaria is controversial, a systematic review and meta-analysis were conducted to examine the variation in GPx blood levels between malaria patients and healthy controls. Additionally, variations in blood levels of GPx in patients with different *Plasmodium* species infections, parasite densities, and various clinical severity levels were evaluated to provide evidence-based data on GPx in malaria.

## Methods

The protocol of this review was published in the International Prospective Register of Systematic Reviews (PROSPERO), number CRD42023421903. The systematic review was conducted according to the recommendation by Cochrane Collaboration^22^. The reports of this systematic review and meta-analysis followed the guideline and standards of the Preferred Reporting Items for Systematic Reviews and Meta-Analyses (PRISMA) statement^23^.

### Search strategy

Six electronic databases, ProQuest, Scopus, Embase, MEDLINE, PubMed, and Ovid, were searched without publication language or publication date limitations. The following search terms were adopted for PubMed: (“Glutathione peroxidase”[Text Word] OR “Selenoglutathione Peroxidase”[Text Word] OR “Glutathione Lipoperoxidase”[Text Word] OR GPx[Text Word] OR “GSH peroxidase”[Text Word] OR GSHPx[Text Word] OR GPxs[Text Word] OR “glutathione peroxidase”[MeSH Terms]) AND (malaria[Text Word] OR Plasmodium[Text Word]). For searches of the five other databases, search terms were adopted slightly from PubMed (Table S1). Additionally, Google Scholar searches were conducted to ensure that all relevant articles had been included and to maximize the number of articles retrieved. Searching in Google Scholar yielded an overwhelmingly high number of results, many of which were irrelevant, so only the first 200 articles were screened for eligibility, per the recommendation made previously^24^. Furthermore, references from the selected articles were evaluated for possible eligibility. The search commenced from the oldest available articles and ended on April 24, 2023.

### Eligibility criteria, study selection, and data extraction

Two reviewers (M.K. and K.U.K.) independently evaluated the eligibility of the studies, and disagreements were resolved through discussion with a third reviewer (A.M.). Studies were included if they fulfilled the following criteria: (i) cross-sectional, clinical trials (with baseline data of outcome), cohort, experimental, or case–control studies, (ii) conducted in *Plasmodium-infected* and uninfected individuals, and (iii) measured GPx levels/activity in both groups of participants. If multiple articles from the same study were found, the article with the most participants was chosen. Two reviewers (M.K. and K.U.K) extracted the following data after deciding the qualified studies to be included: (i) name of the first author, (ii) year of publication, (iii) country, (iv) study design, (v) details of participants, (vi) number of participants, (vii) age of participants, (viii) GPx level/activity, (ix) method for malaria, and (x) method for GPx measurement.

### Certainty of evidence assessment

To evaluate the certainty of the evidence for differing GPx levels between malaria patients and uninfected individuals, the JBI critical appraisal tools for cross-sectional, clinical trials, cohort, experimental, and case–control studies were adopted^25^. These tools assessed the certainty of evidence based on 8, 13, 11, 9, and 10 items for cross-sectional, clinical trials, cohort, experimental, and case–control studies, respectively.

### Statistical analysis

The meta-analysis pooled the effect estimates using a random-effects model with the DerSimonian–Laird method^26^. Outcomes in the meta-analysis were assumed to be Hedges’s g, which is the effect estimate for comparing a treatment’s effect with a control from standardized mean differences (SMDs)^27^. The *I*^2^ statistic and Cochran’s Q test assessed the heterogeneity between studies. A greater homogeneity was regarded as an *I*^2^ value close to zero, whereas *I*^2^ values between 25 and 50% indicated low heterogeneity, 51–75% indicated moderate heterogeneity, and > 75% indicated significant heterogeneity^22^. Meta-regression and subgroup analyses were conducted in the meta-analysis comparing GPx between malaria cases and uninfected controls because it included more than 10 studies and was stratified for publication year, study design, country, continent, *Plasmodium* species, age groups, and clinical status. For comparisons with more than 10 studies, assessment of publication bias and small-study effect were explored by funnel plots, contour-enhanced funnel plots, and Egger’s regression test^28^. All analyses were conducted in Stata version 17.0 (StataCorp LLC, College Station, TX)^29^.

### Sensitivity analysis

The leave-one-out meta-analysis was used to test the impact of individual studies on the pooled effect estimate. Additionally, the fixed effects model was applied for comparisons with the random-effects model to test whether the change of assumption of the statistical model affects the stability and robustness of the results.

## Results

### Search results

**Figure 1:**
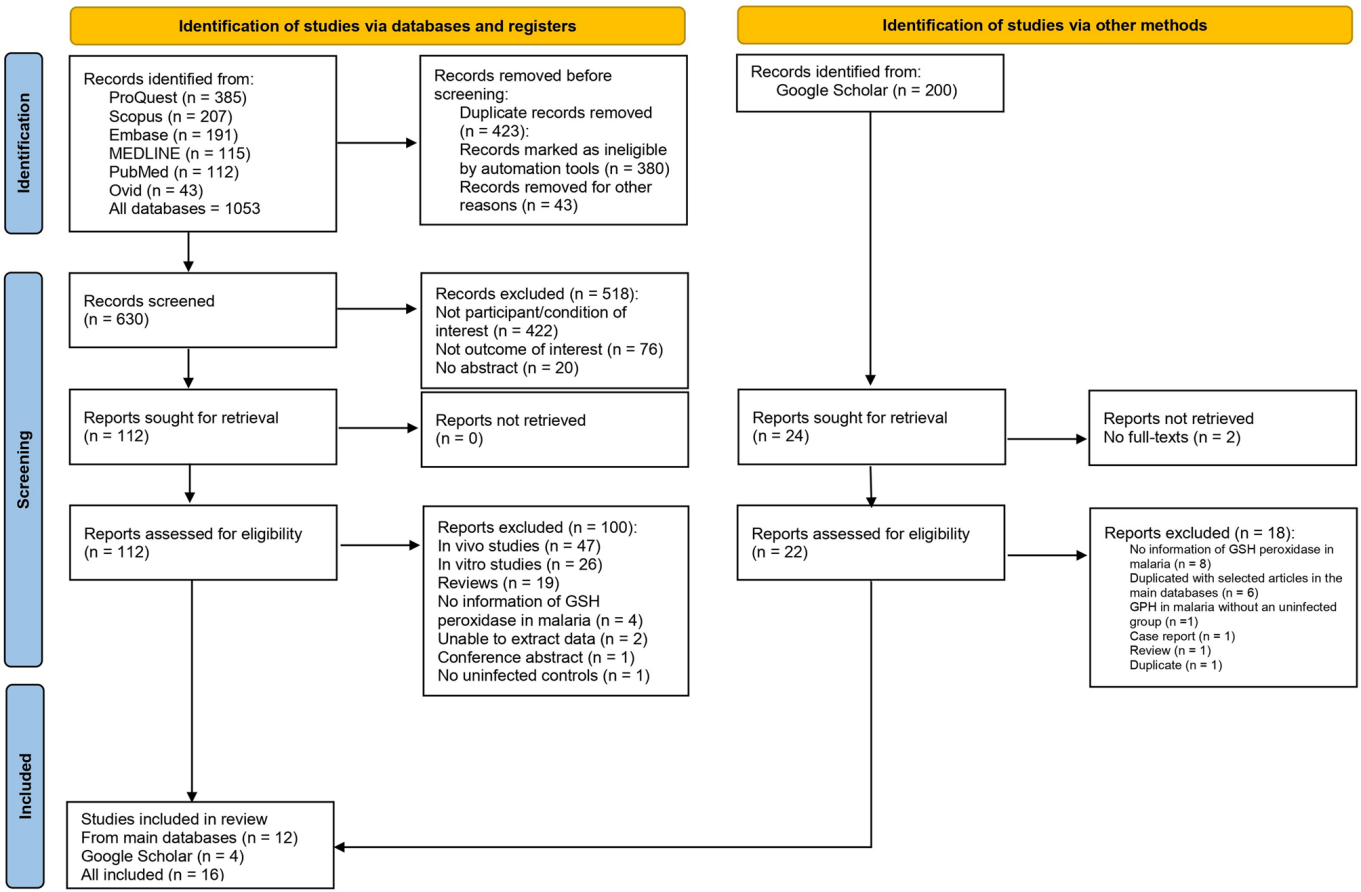
Study flow diagram.

A total of 1,053 articles were retrieved from searches in six databases, including ProQuest (n = 385), Scopus (n = 207), Embase (n = 191), MEDLINE (n = 115), PubMed (n = 112), and Ovid (n = 43). After 423 duplicated articles were removed by automation tools (n = 380) and manual screening (n = 43), the remaining studies (n = 630) were screened according to their relevant titles and abstracts. After non-related articles (n = 518) were excluded, the remaining articles were assessed for eligibility (n = 112). Twelve articles^18,19,21,30–38^ met the eligibility criteria and were included. Further searching in Google Scholar identified four additional studies^20,39–41^ that met the eligibility criteria. No relevant studies were found upon scanning the reference lists of the included studies. Finally, 16 original articles^18–21,30–41^ were included for review (Fig. 1).

### Studies’ characteristics and risk of bias

The majority of studies were published between 2010 and 2023 (75%) and were cross-sectional studies (75%). Most studies were conducted in Africa (56.3%), including Nigeria, Sudan, and Uganda. The remaining studies were conducted in Asia (31.3%), including India and Turkey, Europe (France), and South America (Colombia). Most enrolled individuals infected with *P. falciparum* malaria (62.5%), and most participants were adults (31.3%). Approximately 50% of the participants in the included studies had symptomatic malaria. All included studies used microscopic examination to detect malaria parasites (Tables 1 and S2). The risk of bias among the included studies was examined using the JBI critical appraisal tools for case–control, cohort, cross-sectional, and experimental studies. The results of the assessment showed that one case–control study lacked exposure period of interest^30^; four cross-sectional studies lacked the identification and strategy to deal with confounding factors^19,21,32,40^; and the cohort study had unclear follow-up details^34^. All studies were included in the review (Table S3).

**Table 1:** Characteristics of the studies.

Characteristics	N. (16 studies)	%
**Publication year**		
2010–2023	12	75.0
2000–2009	2	12.5
Before 2000	2	12.5
**Study designs**		
Cross-sectional studies	12	75.0
Cohort study	2	12.5
Case–control studies	1	6.25
Experimental study	1	6.25
**Study areas**		
Africa	9	56.3
Nigeria	7	77.8
Sudan	1	11.1
Uganda	1	11.1
Asia	5	31.3
India	3	60.0
Turkey	2	40.0
Europe (France)	1	6.25
South America (Colombia)	1	6.25
***Plasmodium*** **spp.**		
*P. falciparum*	10	62.5
*P. falciparum, P. vivax*	3	18.8
*P. vivax*	2	12.5
Not specified	1	6.25
**Participants**		
Children	3	18.8
Adults	5	31.3
All age groups	3	18.8
Not specified	5	31.3
**Clinical status**		
Symptomatic malaria	8	50.0
Symptomatic and asymptomatic malaria	2	12.5
Not defined status	6	37.5
**Methods for malaria detection**		
Microscopy	16	100

### GPx between malaria patients and uninfected individuals

Sixteen studies compared blood levels of GPx between malaria patients and uninfected individuals^18–21,30–41^. Based on the finding of these studies, eleven studies demonstrated that GPx blood levels were significantly lower in malaria patients than in uninfected individuals (68.75%)^18,19,30,32–36,39–41^. In contrast, three studies published that blood levels of GPx were significantly higher in malaria patients than in uninfected individuals (18.8%)^20,37,38^. Finally, two studies showed no difference in blood levels of GPx (12.5%)^21,31^.

**Figure 2:**
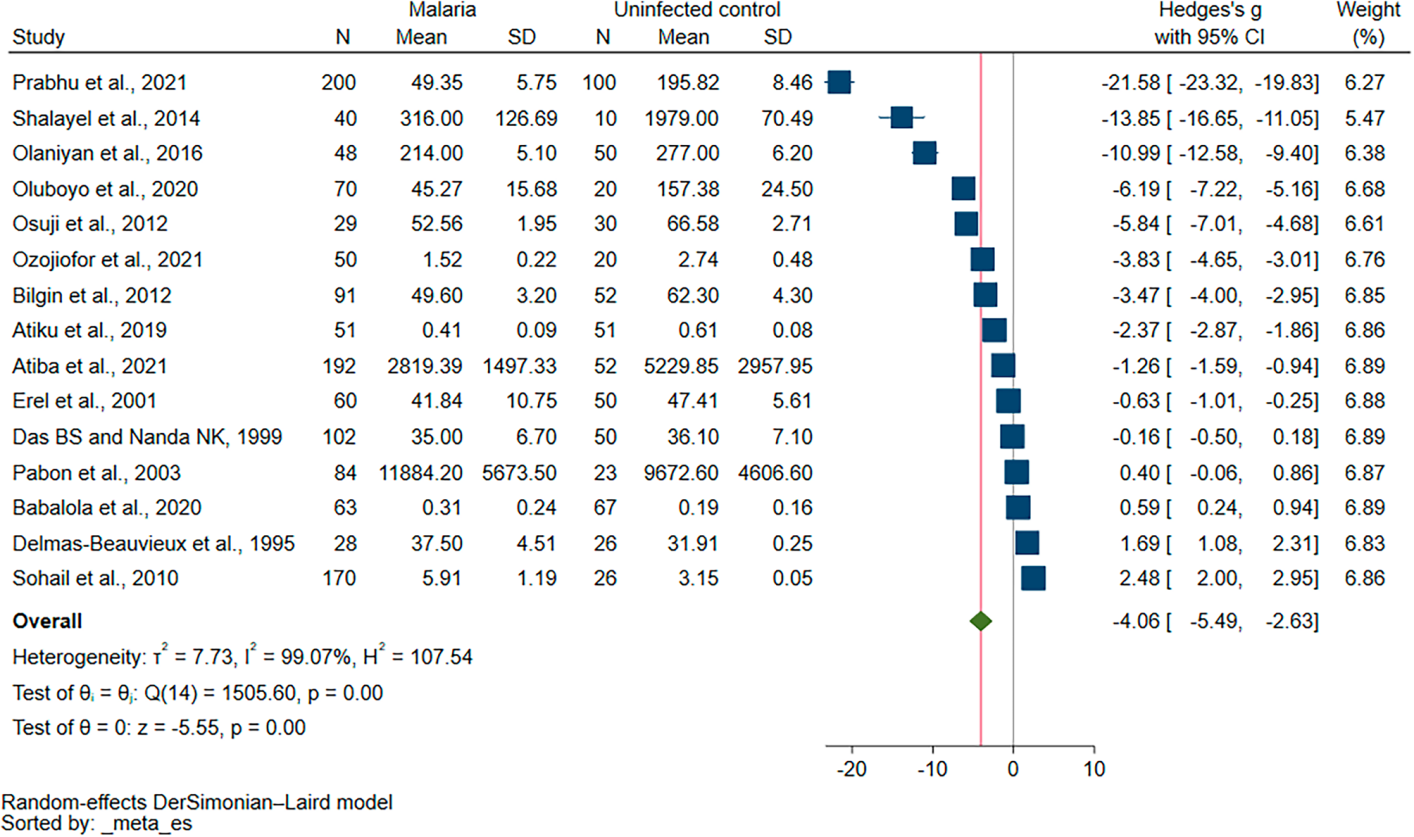
The forest plot demonstrating the difference in GPx levels between malaria patients and uninfected individuals. Abbreviation: CI, confidence interval; Mean Diff., mean difference; N, number of participants; SD, standard deviation.

The difference in blood levels of GPx between malaria patients and uninfected individuals was estimated in the meta-analysis of 15 studies that reported quantitative data^18–21,30–33,35–41^. Results showed diminished blood levels of GPx in malaria patients compared to uninfected individuals (*P* < 0.01, Hedges’ g: − 4.06, 95% CI − 5.49–(− 2.63), *I*^2^: 99.07%, 1,278 malaria patients/627 uninfected individuals, 15 studies, Fig. 2). The meta-regression of publication year, study design, country, continent, *Plasmodium* species, age groups, and clinical status demonstrated that the publication year, *Plasmodium* species, and clinical status significantly affected the pooled estimate (*P* < 0.05, Table S4). Subsequently, subgroup analyses of the publication year, *Plasmodium* species, and clinical status were conducted.

Following subgroup analysis, the publication year had significant subgroup differences (*P* < 0.01, Supplementary Fig. 1). Studies published between 2010 and 2023 showed diminished blood levels of GPx in malaria patients relative to uninfected individuals (*P* < 0.01, Hedges’ g: − 5.83, 95% CI − 7.98–(− 3.69), *I*^2^: 99.26%, 11 studies). In contrast, the blood levels of GPx were similar between the two groups in studies published between 2000 and 2009 (*P* = 0.81, Hedges’ g: − 0.12, 95% CI − 1.14–0.89, *I*^2^: 91.21%, 2 studies), and in studies published before 2000 (*P* = 0.42, Hedges’ g: 0.76, 95% CI − 1.08–2.60, *I*^2^: 96.28%, 2 studies).

Subgroup analysis of the *Plasmodium* species also showed subgroup differences (*P* < 0.01, Supplementary Fig. 2). Studies that enrolled patients with *P. falciparum* infections reported diminished blood levels of GPx in malaria patients compared to uninfected individuals (*P* < 0.01, Hedges’ g: − 3.06, 95% CI − 4.46–(− 1.65), *I*^2^: 98.39%, 9 studies). However, studies that enrolled patients with *P. falciparum* and *P. vivax* malaria reported similar blood levels of GPx between the two groups (*P* = 0.08, Hedges’ g: − 6.31, 95% CI − 12.88–(− 0.63), *I*^2^: 99.71%, 3 studies), and patients with only *P. vivax* malaria (*P* = 0.15, Hedges’ g: − 2.05, 95% CI − 4.83–0.74), *I*^2^: 98.64%, 2 studies).

The subgroup analysis of the clinical status also showed subgroup differences (*P* = 0.01, Supplementary Fig. 3). Studies that enrolled symptomatic malaria patients and did not specify the clinical status of malaria patients demonstrated diminished blood levels of GPx in malaria patients compared to uninfected individuals (*P* = 0.03, Hedges’ g: − 1.55, 95% CI − 2.91–(− 0.18), *I*^2^: 97.99%, 7 studies) and (*P* < 0.01, Hedges’ g: − 8.01, 95% CI − 11.94–(− 4.09), *I*^2^: 99.52%, 6 studies), respectively. However, similar blood levels of GPx between the two groups were shown by studies that enrolled both symptomatic and asymptomatic malaria patients (*P* = 0.42, Hedges’ g: − 2.60, 95% CI − 8.91–3.70, *I*^2^: 99.06%, 2 studies).

### GPx between malaria patients with *P. falciparum* and *P. vivax* infections

**Figure 3:**
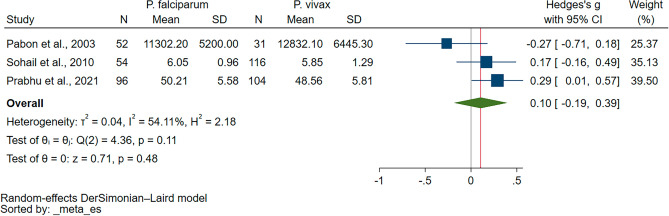
The forest plot indicating the difference in GPx levels between malaria patients *P. vivax* and *P. falcipaum* malaria. Abbreviation: CI, confidence interval; Mean Diff., mean difference; N, number of participants; SD, standard deviation.

Three studies compared blood levels of GPx between malaria patients with *P. vivax* and *P. falciparum* infections^37,38,40^. The findings of the three studies showed similar GPx levels among patients with *P. falciparum* and *P. vivax* malaria^37,38,40^. Following meta-analysis of the three studies, blood levels of GPx were similar between patients with *P. vivax* and *P. falciparum* infections (*P* = 0.48, Hedges’ g: 0.10, 95% CI − 0.19–0.39, *I*^2^: 54.11%, 202 *P. falciparum* patients/251 *P. vivax* patients, 3 studies, Fig. 3).

### GPx between malaria patients with different levels of parasite density and disease severity

Six studies investigated variations in GPx blood levels between malaria patients with various parasite densities^18–21,36,39^. Three studies demonstrated an inverse correlation between blood levels of GPx and parasite density^18,20,36^. Two studies showed no association between blood levels of GPx and parasite density^19,21^. In contrast, one study showed elevated blood levels of GPx in patients with moderate parasite density compared to those with low and high parasite densities^39^.

### Sensitivity analysis

**Figure 4:**
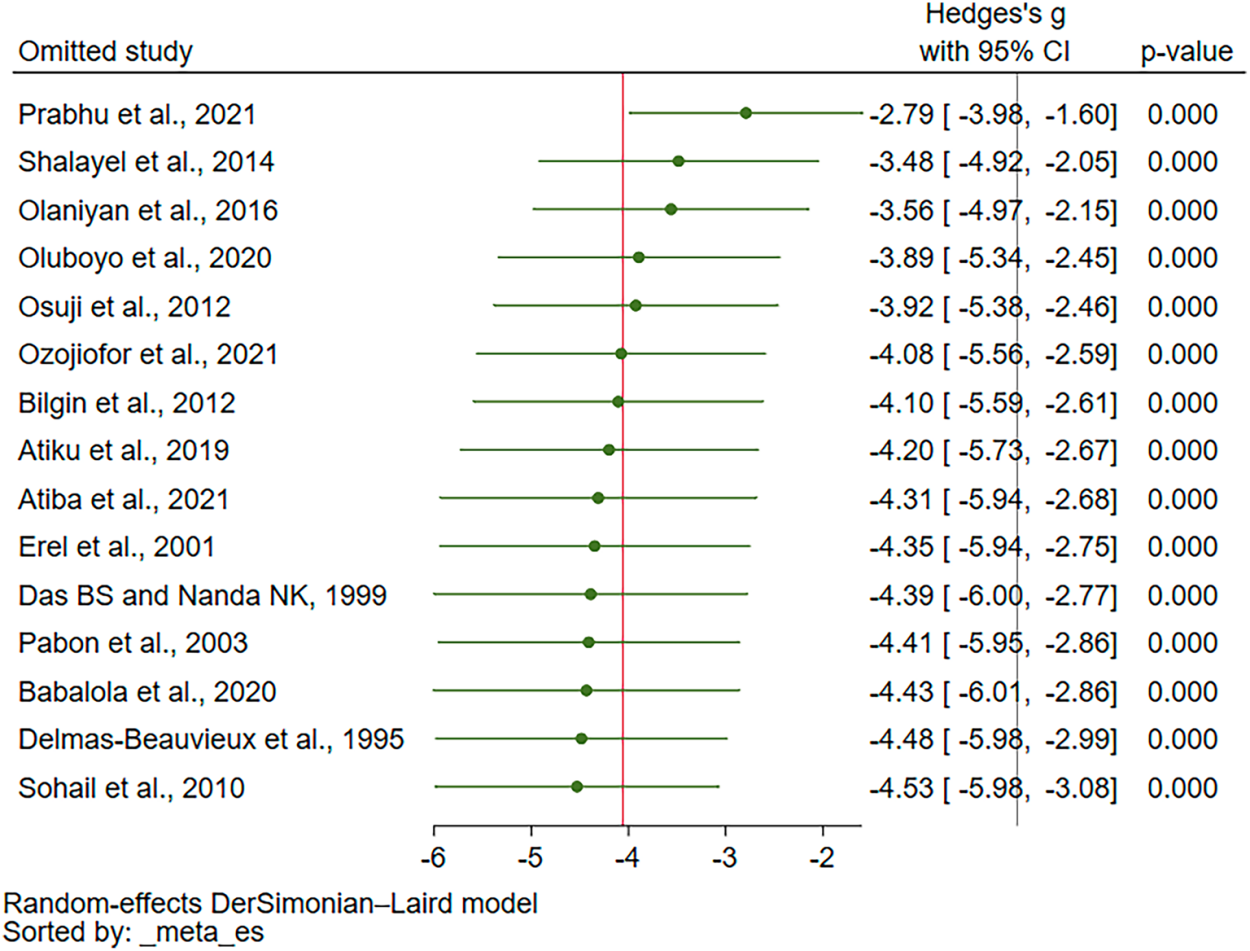
The leave-one-out method showing an outlier in the meta-analysis of the difference in GPx levels between malaria patients and uninfected individuals. Abbreviation: CI, confidence interval.

The leave-one-out meta-analysis showed no impact of individual studies on the pooled effect estimate (*P* value < 0.05 in each rerun meta-analysis, Fig. 4). When the fixed effects model was applied for comparisons with the random-effects model, the results indicated diminished blood levels of GPx in malaria patients compared to uninfected individuals (*P* < 0.01, Hedges’ g: − 0.82, 95% CI − 0.96–(− 0.69), *I*^2^: 99.07%, 15 studies, Supplementary Fig. 4), indicating that the change of assumption of the statistical model did not affect the stability and robustness of the results. The results of the sensitivity analysis indicated that the results of the meta-analysis were robust.

### Publication bias

**Figure 5:**
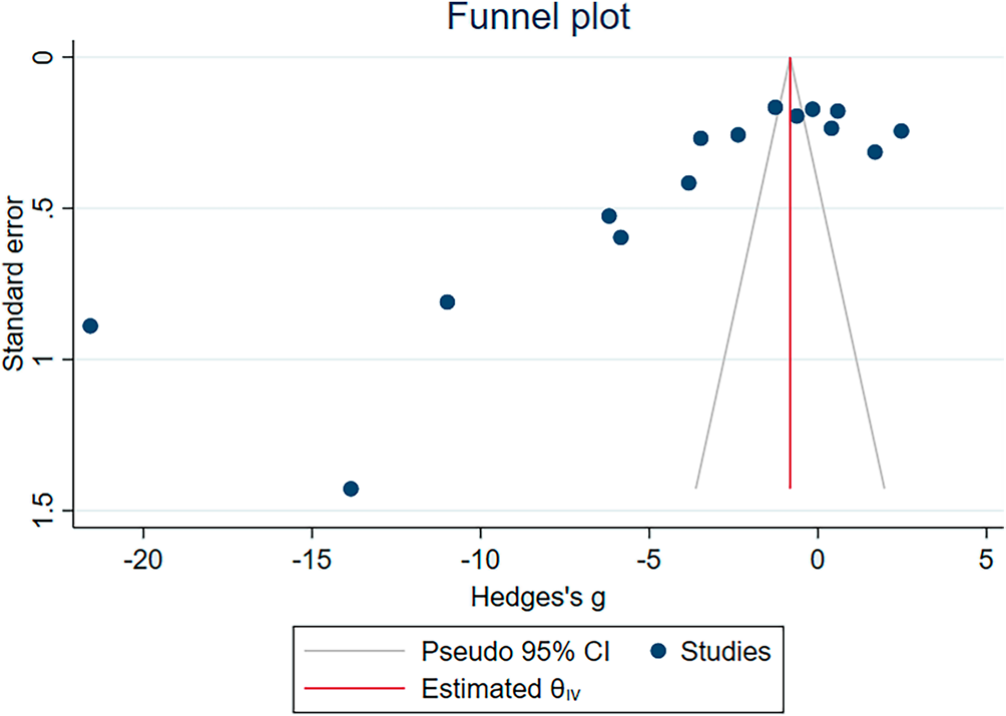
The funnel plot showing an asymmetrical distribution of Hedges’s g (X-axis) and standard error (Y-axis) of GPx levels between malaria patients and uninfected individuals. Abbreviation: CI, confidence interval.

**Figure 6:**
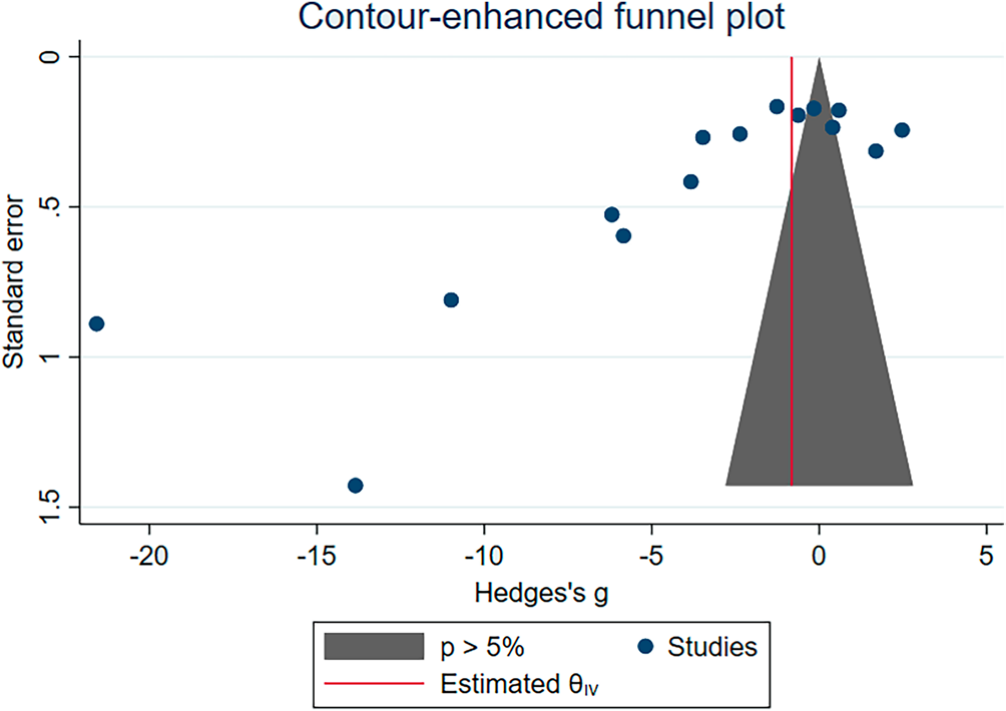
The contour-enhanced funnel plot showing a distribution of Hedges’s g (X-axis) and standard error (Y-axis) of GPx levels between malaria patients and uninfected individuals in significant and non-significant areas of the funnel plot. Abbreviation: CI, confidence interval.

The asymmetrical distribution of Hedges’ g of individual studies was demonstrated by visualization of the funnel plot (Fig. 5). Results of the Egger’s test showed significant differences (*P* < 0.01). The publication bias was due to the small number of studies included in the meta-analysis. In addition, the distribution of Hedges’ g of individual studies was inside the significant area of the Contour-enhanced funnel plot (*P* < 0.05, Fig. 6). Therefore, the heterogeneity of the Hedges’ g from individual studies was the cause of the funnel plot asymmetry.

## Discussion

The present study found decreased blood levels of GPx in malaria patients compared to uninfected individuals. The meta-analysis revealed that both *P. falciparum* and *P. vivax* infections were associated with diminished blood levels of GPx, with no significant difference observed between the two infections. *P. falciparum* generates ROS by breaking down amino acids within the acidic food vacuole. This process can additionally lead to the generation of toxic free-heme (ferri/ferroprotoporphyrin IX; FP) and ROS^8^. The mechanism responsible for the reduced blood levels of GPx in malaria remains unclear, and further research is needed to determine the underlying cause of GPx depletion. Babalola et al. demonstrated that GPx was higher during *P. falciparum* infections^20^. Increased levels of MDA and decreased levels of antioxidant enzymes such as SOD, CAT, and GPx indicate that antioxidant levels are equally increased to combat the generation of oxidative stress products^42,43^.

The subgroup meta-analysis revealed decreased reported GPx levels in studies published between 2010 and 2023. In contrast, comparable GPx levels were observed in studies from before 2009. This discrepancy may stem from differences in study locations or the fact that fewer studies were conducted before 2009 compared to the period between 2010 and 2023. As a result, the conclusion regarding decreased GPx levels between 2010 and 2023 is likely more robust and reliable than conclusions drawn from the earlier studies. Additionally, there were fewer publications on GPx levels conducted before 2009 compared to between 2010 and 2023, which could have contributed to the heterogeneity in the results. The subgroup meta-analysis for the variations in levels of GPx during different *Plasmodium* species infections revealed that a substantial GPx depletion was only seen in patients with falciparum malaria and not in studies that included patients with vivax malaria. However, the direct comparison between patients with falciparum and vivax malaria showed a comparable level of GPx. The possible reason behind this contrary result between subgroup analysis and direct comparison may be explained by the number of studies included in each meta-analysis. Nine studies were included for analysis in the subgroup of *P. falciparum*^18–21,31,35,36,39,41^, but only three studies were included in the direct comparison of GPx between patients with vivax and falciparum malaria^37,38,40^. Regardless of the type of *Plasmodium* infection, both *P. falciparum* or *P. vivax* infection could increase oxidative stress and decrease antioxidant levels. Nevertheless, the meta-analysis results suggest that additional research is required to examine how different *Plasmodium* species infections relate to GPx levels.

The clinical status subgroup analysis revealed that whereas GPx did not change in studies that included symptomatic and asymptomatic malaria patients, it did decrease in studies that only included symptomatic patients. As MDA, a measure of oxidative stress, is considerably greater in symptomatic malaria patients than in asymptomatic malaria patients^20^, people with asymptomatic malaria may exhibit lower levels of oxidative stress. GPx levels were lower in patients with high and moderate parasitemia than in those with low parasitemia. The reason behind the inverse correlation between GPx levels and parasitemia remains unknown. However, because the source of GPx is from the parasites but not the host, the high and moderate parasitemia should correlate positively with levels of GPx. In addition, the correlation between GPx and parasitemia levels may not extend to other antioxidants because the parasite can use antioxidants, including tripeptide GSH, thioredoxin-dependent proteins, and superoxide dismutase as well as other mechanisms to overcome the harmful effects of ROS regardless of the status of GPx^44,45^.

In addition to GPx, which plays a role against oxidative stress-related *Plasmodium* infections, other antioxidants such as SOD, CAT, peroxiredoxin 2 (PRDX2), GSH, ascorbic acid, lipoic acid, α-tocopherol, and β-carotene are necessary to maintain the redox balance^46^. In our previous meta-analysis, levels of SOD in malaria patients were significantly lower compared to uninfected cases^43^. Our previous meta-analysis and this study suggest a possible positive correlation between GPx and SOD. Another antioxidant enzyme is CAT, which ranks among the most significant antioxidants and performs similar functions as GPx and SOD. These three antioxidant enzymes (GPx, SOD, and CAT) directly combat free radicals like peroxynitrite, hydroxyl, and perhydryl radicals to lessen their reactivity^47^. As lipids are the targets for forming oxidative stress, it is hypothesized that hyperlipidemia significantly contributes to the depletion of GPx in the *P. falciparum*-infected erythrocyte^34^. In this regard, anti-malarial drug therapy for malaria patients reduces lipid peroxide levels, which results in significant restoration of antioxidant status, including GSH, SOD, catalase, and GPx levels^34^.

The present study had some limitations. First, the publication bias of the outcome among the included studies may affect the overall effect estimate. Second, the heterogeneity of outcome remained in the subgroup analysis, potentially limiting the conclusion made by the meta-analysis. The difference in GPx levels between *Plasmodium* species, parasite density, and different levels of clinical severity could not be estimated effectively in the present study as only a few studies have investigated this finding.

## Conclusion

In conclusion, malaria is associated with reduced GPx levels. This finding provides valuable insights that prompt the need to investigate the role of GPx depletion in malaria pathogenesis. Further research is necessary to determine the differences in GPx levels between infections caused by various *Plasmodium* species, varying parasite densities, and varying degrees of clinical severity, given the small number of studies included in this meta-analysis. In addition, further studies are required to understand the potential benefits of anti-malarial medications combined with GPx supplementation on the restoration of antioxidant status in varying clinical malaria cases.

### Supplementary Information

Supplementary Figures.

Supplementary Table S1.

Supplementary Table S2.

Supplementary Table S3.

Supplementary Table S4.

## Data Availability

All data relating to the present study are available in this manuscript and supplementary files.
