# Supplementary material for: Impact of malaria on glutathione peroxidase levels: a systematic review and meta-analysis
Source: Sci Rep. 2023 Aug 25;13:13928. doi: 10.1038/s41598-023-41056-x (PMC10457399; doi:10.1038/s41598-023-41056-x)
Supplement: Supplementary file 1 — Supplementary Figures. [file 41598_2023_41056_MOESM1_ESM.pdf]

Impact of malaria on glutathione peroxidase levels: A systematic review and meta-analysis

Running title: Glutathione peroxidase in malaria patients

Manas Kotepui[1], Aongart Mahittikorn[2*], Nsoh Godwin Anabire[3,4], Kwuntida Uthaisar Kotepui[1]*

[1]Medical Technology, School of Allied Health Sciences, Walailak University, Tha Sala, Nakhon Si Thammarat, Thailand

[2]Department of Protozoology, Faculty of Tropical Medicine, Mahidol University, Bangkok, Thailand

[3]Department of Biochemistry & Molecular Medicine, School of Medicine, University for Development Studies, Tamale, Ghana.

[4]West African Centre for Cell Biology of Infectious Pathogens (WACCBIP); Department of Biochemistry, Cell & Molecular Biology, University of Ghana, Accra, Ghana.

[*]**Corresponding author**

Manas Kotepui :manas.ko@wu.ac.th

Aongart Mahittikorn: aongart.mah@mahidol.ac.th

Nsoh Godwin Anabire: nanabire@uds.edu.gh

Kwuntida Uthaisar Kotepui :kwuntida.ut@wu.ac.th

Supplementary Figures

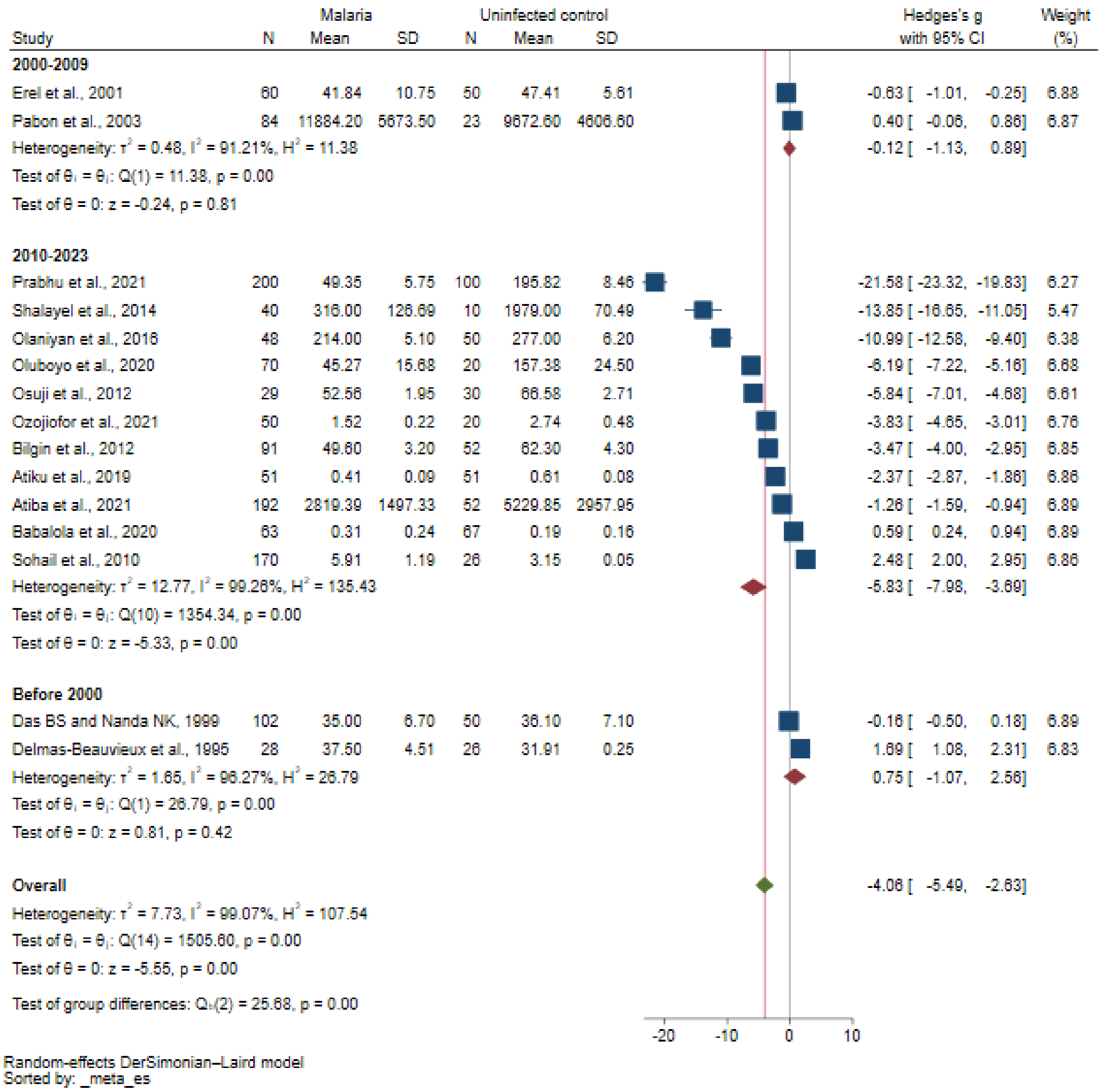

Supplementary Figure 1. The forest plot showing the difference in GPx levels between malaria patients and uninfected individuals stratified by publication years. Abbreviation: CI, confidence interval; Mean Diff., mean difference; N, number of participants; SD, standard deviation.

Study	Malaria N	Malaria Mean	Malaria SD	Uninfected control N	Uninfected control Mean	Uninfected control SD	Hedges's g with 95% CI	Weight (%)
Prabhu et al., 2021	200	49.35	5.75	100	195.82	8.46	-21.58 [-23.32, -19.83]	6.27
Shalayel et al., 2014	40	316.00	126.69	10	1979.00	70.49	-13.85 [-16.65, -11.05]	5.47
Olaniyan et al., 2016	48	214.00	5.10	50	277.00	6.20	-10.99 [-12.58, -9.40]	6.38
Oluboyo et al., 2020	70	45.27	15.68	20	157.38	24.50	-6.19 [-7.22, -5.16]	6.68
Osuji et al., 2012	29	52.56	1.95	30	66.58	2.71	-5.84 [-7.01, -4.68]	6.61
Ozojiofor et al., 2021	50	1.52	0.22	20	2.74	0.48	-3.83 [-4.65, -3.01]	6.76
Bilgin et al., 2012	91	49.60	3.20	52	62.30	4.30	-3.47 [-4.00, -2.95]	6.85
Atiku et al., 2019	51	0.41	0.09	51	0.61	0.08	-2.37 [-2.87, -1.86]	6.86
Atiba et al., 2021	192	2819.39	1497.33	52	5229.85	2957.95	-1.26 [-1.59, -0.94]	6.89
Erel et al., 2001	60	41.84	10.75	50	47.41	5.61	-0.63 [-1.01, -0.25]	6.88
Das BS and Nanda NK, 1999	102	35.00	6.70	50	36.10	7.10	-0.16 [-0.50, 0.18]	6.89
Pabon et al., 2003	84	11884.20	5673.50	23	9672.60	4606.60	0.40 [-0.06, 0.86]	6.87
Babalola et al., 2020	63	0.31	0.24	67	0.19	0.16	0.59 [0.24, 0.94]	6.89
Delmas-Beauvieux et al., 1995	28	37.50	4.51	26	31.91	0.25	1.69 [1.08, 2.31]	6.83
Sohail et al., 2010	170	5.91	1.19	26	3.15	0.05	2.48 [2.00, 2.95]	6.86
**Overall**							-4.06 [-5.49, -2.63]	

Heterogeneity: $\tau^2 = 7.73$, $I^2 = 99.07\%$, $H^2 = 107.54$

Test of $\theta_i = \theta_j$: Q(14) = 1505.60, p = 0.00

Test of $\theta = 0$: z = -5.55, p = 0.00

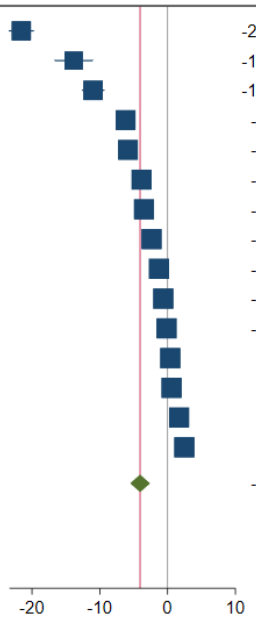

Random-effects DerSimonian–Laird model
Sorted by: _meta_es

Supplementary Figure 2. The forest plot showing the difference in GPx levels between malaria patients and uninfected individuals stratified by *Plasmodium* species. Abbreviation: CI, confidence interval; Mean Diff., mean difference; N, number of participants; SD, standard deviation.

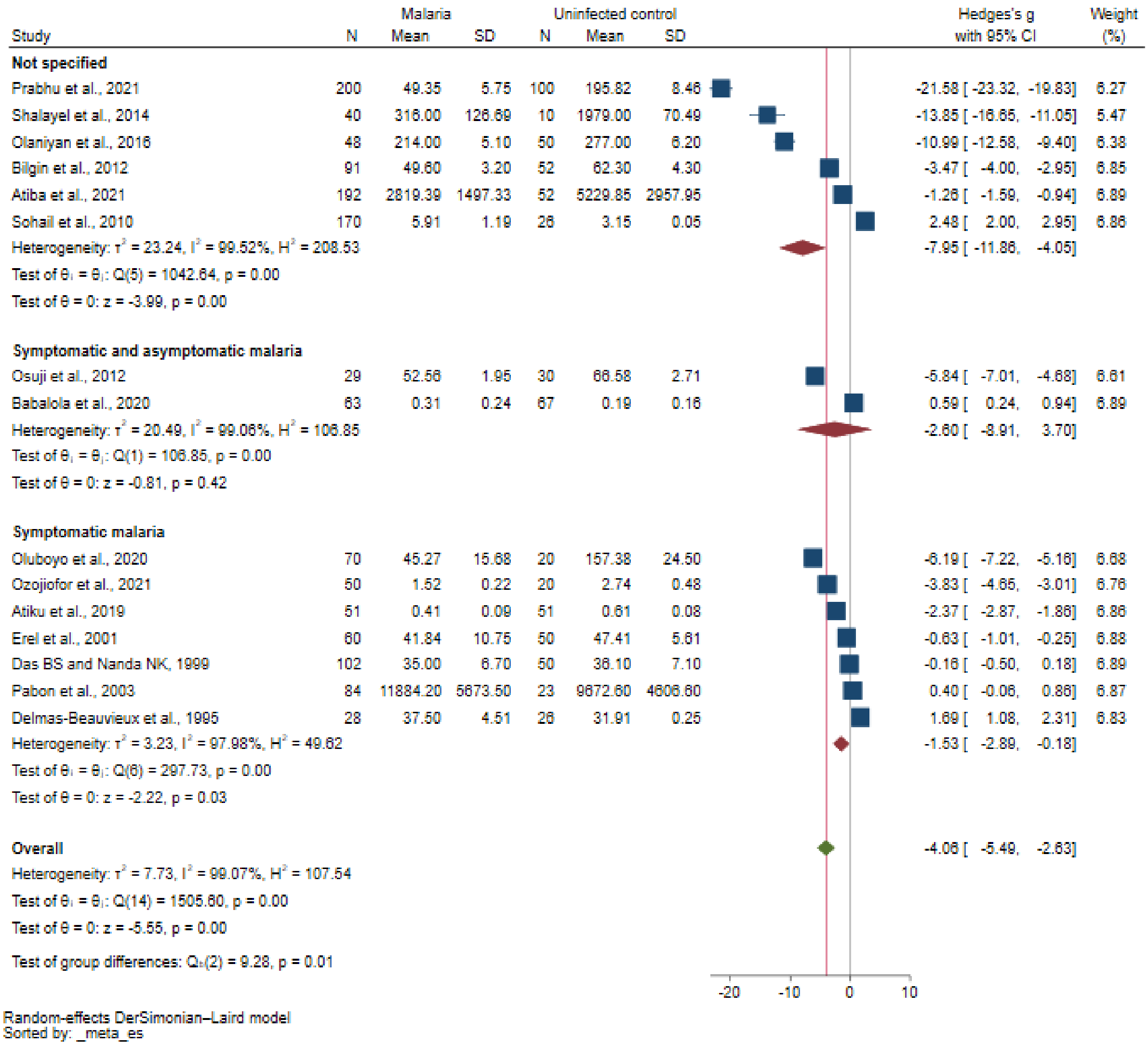

Supplementary Figure 3. The forest plot showing the difference in GPx levels between malaria patients and uninfected individuals stratified by clinical status. Abbreviation: CI, confidence interval; Mean Diff., mean difference; N, number of participants; SD, standard deviation.

Study	Malaria N	Malaria Mean	Malaria SD	Uninfected control N	Uninfected control Mean	Uninfected control SD	Hedges's g with 95% CI	Weight (%)
Prabhu et al., 2021	200	49.35	5.75	100	195.82	8.46	-21.58 [-23.32, -19.83]	0.58
Shalayel et al., 2014	40	316.00	126.69	10	1979.00	70.49	-13.85 [-16.65, -11.05]	0.23
Olaniyan et al., 2016	48	214.00	5.10	50	277.00	6.20	-10.99 [-12.58, -9.40]	0.70
Oluboyo et al., 2020	70	45.27	15.68	20	157.38	24.50	-6.19 [-7.22, -5.16]	1.67
Osuji et al., 2012	29	52.56	1.95	30	66.58	2.71	-5.84 [-7.01, -4.68]	1.29
Ozojiofor et al., 2021	50	1.52	0.22	20	2.74	0.48	-3.83 [-4.65, -3.01]	2.66
Bilgin et al., 2012	91	49.60	3.20	52	62.30	4.30	-3.47 [-4.00, -2.95]	6.38
Atiku et al., 2019	51	0.41	0.09	51	0.61	0.08	-2.37 [-2.87, -1.86]	6.96
Atiba et al., 2021	192	2819.39	1497.33	52	5229.85	2957.95	-1.26 [-1.59, -0.94]	16.69
Erel et al., 2001	60	41.84	10.75	50	47.41	5.61	-0.63 [-1.01, -0.25]	12.12
Das BS and Nanda NK, 1999	102	35.00	6.70	50	36.10	7.10	-0.16 [-0.50, 0.18]	15.55
Pabon et al., 2003	84	11884.20	5673.50	23	9672.60	4606.60	0.40 [-0.06, 0.86]	8.31
Babalola et al., 2020	63	0.31	0.24	67	0.19	0.16	0.59 [0.24, 0.94]	14.48
Delmas-Beauvieux et al., 1995	28	37.50	4.51	26	31.91	0.25	1.69 [1.08, 2.31]	4.67
Sohail et al., 2010	170	5.91	1.19	26	3.15	0.05	2.48 [2.00, 2.95]	7.72
**Overall**							-0.82 [-0.96, -0.69]	

Heterogeneity: I^2 = 99.07%, H^2 = 107.54

Test of $\theta_i = \theta_j$: Q(14) = 1505.60, p = 0.00

Test of θ = 0: z = -12.15, p = 0.00

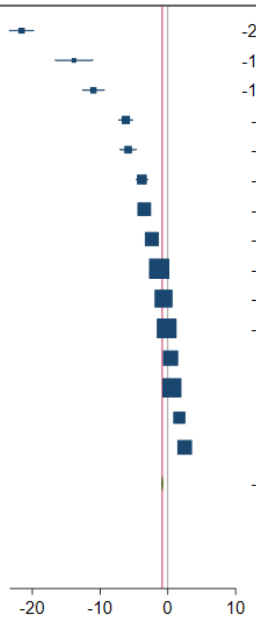

Fixed-effects inverse-variance model
Sorted by: _meta_es

Supplementary Figure 4. The forest plot showing the difference in GPx levels between malaria patients and uninfected individuals by the fixed-effect model. Abbreviation: CI, confidence interval; Mean Diff., mean difference; N, number of participants; SD, standard deviation.
