# Supplementary material for: Impact of malaria on glutathione peroxidase levels: a systematic review and meta-analysis
Source: Sci Rep. 2023 Aug 25;13:13928. doi: 10.1038/s41598-023-41056-x (PMC10457399; doi:10.1038/s41598-023-41056-x)
Supplement: Supplementary file 2 — Supplementary Table S1. [file 41598_2023_41056_MOESM2_ESM.docx]

**Impact of malaria on glutathione peroxidase levels: A systematic review and meta-analysis**

**Running title:** Glutathione peroxidase in malaria patients

Manas Kotepui^1^, Aongart Mahittikorn^2*^, Nsoh Godwin Anabire^3,4^, Kwuntida Uthaisar Kotepui^1^*

^1^Medical Technology, School of Allied Health Sciences, Walailak University, Tha Sala, Nakhon Si Thammarat, Thailand

^2^Department of Protozoology, Faculty of Tropical Medicine, Mahidol University, Bangkok, Thailand

^3^Department of Biochemistry & Molecular Medicine, School of Medicine, University for Development Studies, Tamale, Ghana.

^4^West African Centre for Cell Biology of Infectious Pathogens (WACCBIP); Department of Biochemistry, Cell & Molecular Biology, University of Ghana, Accra, Ghana.

**^*^Corresponding author**

Manas Kotepui: manas.ko@wu.ac.th

Aongart Mahittikorn: [aongart.mah@mahidol.ac.th](mailto:aongart.mah@mahidol.ac.th)

Nsoh Godwin Anabire: [nanabire@uds.edu.gh](mailto:nanabire@uds.edu.gh)

Kwuntida Uthaisar Kotepui: [kwuntida.ut@wu.ac.th](mailto:kwuntida.ut@wu.ac.th)

**Table S1. Search terms**

PubMed 19 April 2023

| Key concept | No. | Search terms | Results |
| --- | --- | --- | --- |
| Glutathione peroxidase | 1. | "Glutathione peroxidase"[Text Word] | 41420 |
|  | 2. | "Selenoglutathione Peroxidase"[Text Word] | 7 |
|  | 3. | "Glutathione Lipoperoxidase"[Text Word] | 3 |
|  | 4. | GPx[Text Word] | 14276 |
|  | 5. | "GSH peroxidase"[Text Word] | 800 |
|  | 6. | GSHPx[Text Word] | 8221 |
|  | 7. | GPxs[Text Word] | 2019 |
|  | 8. | "glutathione peroxidase"[MeSH Terms] | 21244 |
|  | 9. | #1 OR #2 OR #3 OR #4 OR #5 OR #6 OR #7 OR #8 | 46667 |
| Malaria | 10. | malaria[Text Word] | 104822 |
|  | 11. | plasmodium[Text Word] | 66786 |
|  | 12. | #10 OR #11 | 117541 |
| Glutathione peroxidase AND Malaria | 13. | #9 AND #12 | 112 |

Embase 24 April 2023

| No. | Key concept | Search terms | Results |
| --- | --- | --- | --- |
| 1. | Glutathione peroxidase | ‘Glutathione peroxidase’:ti,ab,kw,de OR ‘Selenoglutathione Peroxidase’:ti,ab,kw,de OR ‘Glutathione Lipoperoxidase’:ti,ab,kw,de  OR GPx:ti,ab,kw,de OR ‘GSH peroxidase’:ti,ab,kw,de OR GSHPx:ti,ab,kw,de OR GPxs:ti,ab,kw,de OR ‘Selenoglutathione Peroxidase’:ti,ab,kw,de OR ‘Glutathione Lipoperoxidase’:ti,ab,kw,de OR ‘glutathione hydrogen peroxide oxidoreductase’ti,ab,kw,de OR ‘glutathione peroxydase’ti,ab,kw,de OR ‘GSH Px protein’’ti,ab,kw,de OR ‘protein GSH Px’ti,ab,kw,de OR ‘glutathione peroxidase’ti,ab,kw,de OR ‘glutathion peroxidase’/exp | 69656 |
| 2. | Malaria | malaria:ti,ab,kw,de OR plasmodium:ti,ab,kw,de OR ‘Remittent Fever’:ti,ab,kw,de OR ‘Marsh Fever’:ti,ab,kw,de OR Paludism:ti,ab,kw,de OR malaria/exp | 155232 |
| 3. | 1 AND 2 | (‘Glutathione peroxidase’:ti,ab,kw,de OR ‘Selenoglutathione Peroxidase’:ti,ab,kw,de OR ‘Glutathione Lipoperoxidase’:ti,ab,kw,de  OR GPx:ti,ab,kw,de OR ‘GSH peroxidase’:ti,ab,kw,de OR GSHPx:ti,ab,kw,de OR GPxs:ti,ab,kw,de OR ‘Selenoglutathione Peroxidase’:ti,ab,kw,de OR ‘Glutathione Lipoperoxidase’:ti,ab,kw,de OR ‘glutathione hydrogen peroxide oxidoreductase’ti,ab,kw,de OR ‘glutathione peroxydase’ti,ab,kw,de OR ‘GSH Px protein’’ti,ab,kw,de OR ‘protein GSH Px’ti,ab,kw,de OR ‘glutathione peroxidase’ ti,ab,kw,de OR ‘glutathion peroxidase’/exp) AND (malaria:ti,ab,kw,de OR plasmodium:ti,ab,kw,de OR ‘Remittent Fever’:ti,ab,kw,de OR ‘Marsh Fever’:ti,ab,kw,de OR Paludism:ti,ab,kw,de OR malaria/exp) | 191 |

Scopus 24 April 2023

| No. | Key concept | Search terms | Results |
| --- | --- | --- | --- |
| 1. | Glutathione peroxidase | TITLE-ABS-KEY ( "glutathione peroxidase" OR "selenoglutathione peroxidase" OR "glutathione lipoperoxidase" OR gpx OR "gsh peroxidase" OR gshpx OR gpxs OR "selenoglutathione peroxidase" OR "glutathione lipoperoxidase" ) | 73132 |
| 2. | Malaria | TITLE-ABS-KEY ( malaria OR plasmodium OR "plasmodium infection" OR "remittent fever" OR "marsh fever" OR paludism ) | 155983 |
| 3. | 1 AND 2 | ( TITLE-ABS-KEY ( "Glutathione peroxidase" OR "Selenoglutathione Peroxidase" OR "Glutathione Lipoperoxidase" OR gpx OR "GSH peroxidase" OR gshpx OR gpxs OR "Selenoglutathione Peroxidase" OR "Glutathione Lipoperoxidase" ) ) AND ( TITLE-ABS-KEY ( malaria OR plasmodium OR "Plasmodium Infection" OR "Remittent Fever" OR "Marsh Fever" OR paludism ) ) | 207 |

MEDLINE 24 April 2023

| No. | Key concept | Search terms | Results |
| --- | --- | --- | --- |
| 1. | Glutathione peroxidase AND Malaria | (“Glutathione peroxidase” OR “Selenoglutathione Peroxidase” OR “Glutathione Lipoperoxidase” OR GPx OR “GSH peroxidase” OR GSHPx OR GPxs OR ”Selenoglutathione Peroxidase” OR ”Glutathione Lipoperoxidase”) AND (malaria OR plasmodium OR “Plasmodium Infection“ OR “Remittent Fever“ OR “Marsh Fever“ OR Paludism) | 115 |

Ovid 24 April 2023

| No. | Key concept | Search terms | Results |
| --- | --- | --- | --- |
| 1. | Glutathione peroxidase AND Malaria | (“Glutathione peroxidase” OR “Selenoglutathione Peroxidase” OR “Glutathione Lipoperoxidase” OR GPx OR “GSH peroxidase” OR GSHPx OR GPxs OR ”Selenoglutathione Peroxidase” OR ”Glutathione Lipoperoxidase”) AND (malaria OR plasmodium OR “Plasmodium Infection“ OR “Remittent Fever“ OR “Marsh Fever“ OR Paludism) {No Related Terms}  limit to (ovid full text available and articles with abstracts and original articles) | 43 |

ProQuest 24 April 2023

| No. | Key concept | Search terms | Results |
| --- | --- | --- | --- |
| 1. | Glutathione peroxidase AND Malaria | (“Glutathione peroxidase” OR “Selenoglutathione Peroxidase” OR “Glutathione Lipoperoxidase” OR GPx OR “GSH peroxidase” OR GSHPx OR GPxs OR ”Selenoglutathione Peroxidase” OR ”Glutathione Lipoperoxidase”) AND (malaria OR plasmodium OR “Plasmodium Infection“ OR “Remittent Fever“ OR “Marsh Fever“ OR Paludism) | 385 |

Google Scholar 24 April 2023

| No. | Key concept | Search terms | Results |
| --- | --- | --- | --- |
| 1. | Glutathione peroxidase AND Malaria | (“Glutathione peroxidase” OR “Selenoglutathione Peroxidase” OR “Glutathione Lipoperoxidase” OR GPx OR “GSH peroxidase” OR GSHPx OR GPxs OR ”Selenoglutathione Peroxidase” OR ”Glutathione Lipoperoxidase”) AND (malaria OR plasmodium OR “Plasmodium Infection“ OR “Remittent Fever“ OR “Marsh Fever“ OR Paludism) | Limited the screening to the first 200 articles |
