# Supplementary material for: Impact of malaria on glutathione peroxidase levels: a systematic review and meta-analysis
Source: Sci Rep. 2023 Aug 25;13:13928. doi: 10.1038/s41598-023-41056-x (PMC10457399; doi:10.1038/s41598-023-41056-x)
Supplement: Supplementary file 5 — Supplementary Table S4. [file 41598_2023_41056_MOESM5_ESM.docx]

**Impact of malaria on glutathione peroxidase levels: A systematic review and meta-analysis**

**Running title:** Glutathione peroxidase in malaria patients

Manas Kotepui^1^, Aongart Mahittikorn^2*^, Nsoh Godwin Anabire^3,4^, Kwuntida Uthaisar Kotepui^1^*

^1^Medical Technology, School of Allied Health Sciences, Walailak University, Tha Sala, Nakhon Si Thammarat, Thailand

^2^Department of Protozoology, Faculty of Tropical Medicine, Mahidol University, Bangkok, Thailand

^3^Department of Biochemistry & Molecular Medicine, School of Medicine, University for Development Studies, Tamale, Ghana.

^4^West African Centre for Cell Biology of Infectious Pathogens (WACCBIP); Department of Biochemistry, Cell & Molecular Biology, University of Ghana, Accra, Ghana.

**^*^Corresponding author**

Manas Kotepui: manas.ko@wu.ac.th

Aongart Mahittikorn: [aongart.mah@mahidol.ac.th](mailto:aongart.mah@mahidol.ac.th)

Nsoh Godwin Anabire: [nanabire@uds.edu.gh](mailto:nanabire@uds.edu.gh)

Kwuntida Uthaisar Kotepui: [kwuntida.ut@wu.ac.th](mailto:kwuntida.ut@wu.ac.th)

**Table S4. Meta-regression results**

| **Meta-analysis of GPx** | **Covariates** | **P value** | **tau2** | **I^2^ (%)** |
| --- | --- | --- | --- | --- |
| **Malaria patients vs uninfected individuals** | Publication years | < 0.01 | 9.703 | 99.14 |
|  | Study design | 0.07 | 8.445 | 99.11 |
|  | Continent | 0.09 | 9.842 | 99.19 |
|  | Age group | 0.51 | 12.67 | 99.24 |
|  | *Plasmodium* species | 0.04 | 8.294 | 99.12 |
|  | Clinical status | < 0.01 | 10.24 | 99.17 |
